# Recent Progress in Surface-Enhanced Raman Scattering for the Detection of Chemical Contaminants in Water

**DOI:** 10.3389/fchem.2020.00478

**Published:** 2020-06-09

**Authors:** Gustavo Bodelón, Isabel Pastoriza-Santos

**Affiliations:** ^1^CINBIO, University of Vigo, Vigo, Spain; ^2^Galicia Sur Health Research Institute (IIS Galicia Sur) SERGAS-UVIGO, Vigo, Spain

**Keywords:** plasmonic nanostructures, SERS, sensing, chemical contaminants, water

## Abstract

Water is a matter of vital importance for all developed countries due to the strong impact on human health and aquatic, wetlands and terrestrial environments. Therefore, the monitoring of water quality is of tremendous importance. The enormous advantages that Surface-enhanced Raman scattering (SERS) spectroscopy offers, such as fingerprint recognition, multiplex capabilities, high sensitivity, and selectivity or non-destructive testing, make this analytical tool very attractive for this purpose. This minireview aims to provide a summary of current approaches for the implementation of SERS sensors in monitoring organic and inorganic pollutants in water. In addition, we briefly highlight current challenges and provide an outlook for the application of SERS in environmental monitoring.

## Introduction

In recent years, water quality has become a critical concern of most developed countries due to the strong impact on human health and aquatic, wetlands, and terrestrial environments. The growth of human populations, the expansion of industrial and agricultural activities and climate change have been identified as the main threats to cause declining water quality. Therefore, actions for detection, identification, and quantification of pollutants and toxins in water are urgently required. Recently, different agencies such as the European Environment Agency (EEA) or Environmental Protection Agency (EPA, USA) have established legal obligations to protect and restore the quality of water. For instance, the Water Framework Directive (WFD) is the most substantial and ambitious piece of legislation dealing with the protection, monitoring, and management of water quality (European Commission, Introduction to the New EU Water Framework Directive, 2016). For WFD backed up by REACH regulation, which defines the chemical status by environmental quality standards of 41 priority substances.

Scientific findings show that major water pollutants are complex mixtures of chemicals of different categories (biocides, pharmaceuticals and industrial chemicals, pesticides, etc.). Analytical determination of these pollutants is typically carried out by sampling, extraction, and separation of the chemical compounds from the aqueous matrix by high-performance liquid chromatography or gas chromatography, coupled to selective detection methods such as modern mass spectrometry techniques. Generally speaking, these methods have high sensitivity, good specificity, and outstanding precision. However, all of them require complex equipment and laborious operations, which may lead to inaccurate results because the water samples may undergo chemical and physical transformations. Also, there is an urgent requirement for sensitive detection methods which are simpler and portable for on-site analysis. Thus, the development of improved systems for environmental analysis has attracted a high interest in industry and the research community. Among them, surface-enhanced Raman scattering (SERS) has been extensively applied in various types of ultrasensitive chemical detection in a wide variety of fields (Langer et al., 2019). In SERS, the excitation of localized surface plasmon resonances supported by metal nanostructured leads to a massive intensification of the Raman scattering from molecules adsorbed or located in close proximity to the metallic surface (Schlücker, 2014). This effect has resulted in an ultrasensitive plasmon-enhanced spectroscopic technique, which retains the intrinsic structural specificity, as well as the experimental flexibility of Raman spectroscopy. Owing to continuous advances in nanofabrication techniques facilitating the engineering of rationally design plasmonic nanomaterials (Mosier-Boss, 2017; Hamon and Liz-Marzan, 2018; Langer et al., 2019), SERS is progressively expanding into the realm of viable detection of environmental pollutants, as it has been recently reviewed elsewhere (Jiang et al., 2018; Shi et al., 2018; Tang et al., 2018; Choi et al., 2019; Song et al., 2019).

This minireview intends to provide an overview of current approaches undertaken for the implementation of SERS sensors in monitoring organic and inorganic pollutants in water (Supplementary Table 1). Moreover, we aim to reveal the importance and potential of SERS technology for the ultradetection of pollutants in aqueous samples. Finally, a brief challenges and outlook section has been included. Nevertheless, it is out of the scope of this minireview the description of the theory behind SERS or the discussion of the different categories of SERS substrates or methodologies.

## SERS detection of Organic Pollutants

Organic pollutants include many herbicides and insecticides from the agriculture sector, other molecules manufactured for use in various industries [phthalates, polychlorinated byphenyls (PCBs)], by-products of natural or artificial processes [such us, polycyclic aromatic hydrocarbons (PAH), dioxin, etc.], among others.

**Figure 1:**
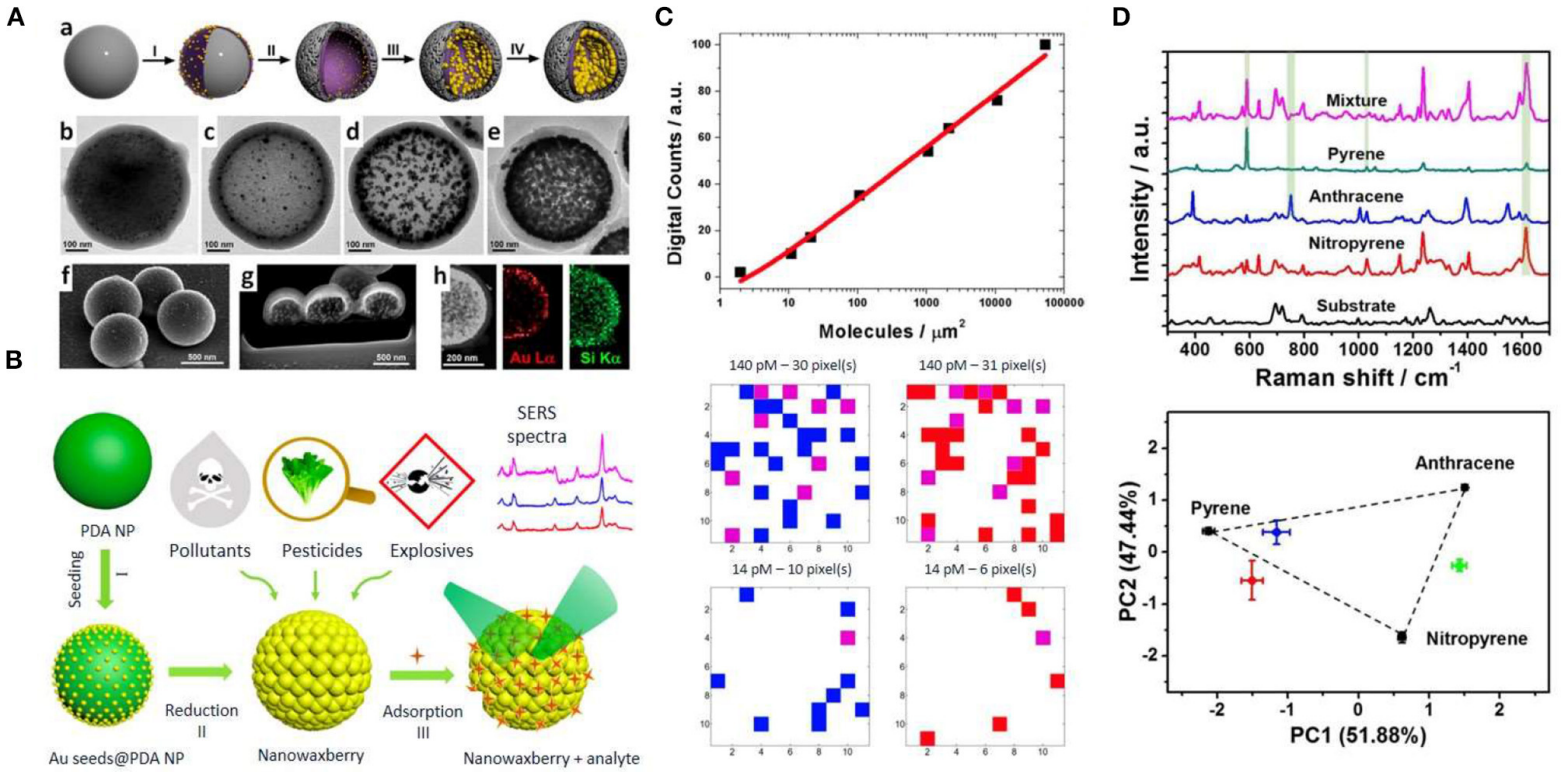
Strategies for SERS detection of organic pollutants. **(A)** Microporous plasmonic capsules. (a) Schematic illustration of the capsules fabrication. (b–e) TEM characterization of the Au NPs growth within the capsules. (f–h) SEM characterization and elemental analysis (h) of the microporous plasmonic capsules. Reproduced from Marino-Lopez et al. (2019) with permission from John Wiley & Sons (Copyright 2019). **(B)** Schematic illustration of the synthesis of nanowaxberry (I-II), and analyte adsorption and SERS detection (III). Reproduced from Chen et al. (2018) with permission from American Chemical Society (Copyright 2018). **(C)** Digital calibration curve generated at ultralow concentration of enrofloxacin (upper panel) and SERS digital mappings obtained from mixtures of two isotopologues of ciprofloxacin. Reproduced from de Albuquerque et al. (2018) with permission from American Chemical Society (Copyright 2018). **(D)** SERS-based multiplex detection of PAHs using pillar[5]arene-based supramolecular plasmonic thin films: Characteristic SERS spectra of pyrene, anthracene, nitropyrene as well as of a mixture of the three PAHs (upper panel) and principal component analysis (PCA) score plot of the first three PCs modeled by the SERS spectra pyrene, anthracene, and nitropyrene (black dots) and their binary mixtures. Reproduced from Montes-Garcia et al. (2017) with permission from American Chemical Society (Copyright 2017).

Direct SERS analysis in natural and contaminated waters is often impaired by the non-specific co-adsorption onto metallic nanostructures of other species in the matrix solution. This can significantly increase the complexity of the vibrational assignment or even completely prevent the interaction with the target analyte, thereby decreasing the sensitivity of the detection assay. In order to circumvent these issues, Marino-Lopez et al. (2019) developed a SERS substrate based on a microporous silica capsule with gold nanoparticles (NPs) in the interior (see Figure 1A). The microporous structure acts as molecular sieving avoiding large biomolecules and cells from reaching the plasmonic component while imparting colloidal stability. The applicability for environmental analysis was demonstrated using river water spiked with dichlorodiphenyltrichloroethane (DDT), a pesticide classified as a persistent organic pollutant and a probable human carcinogen. A limit of detection (LOD) of 1.77 μg/L was reached with this sensing platform. Wang et al. (2019) fabricated a ternary film-packaged bimetallic Au/Ag chip as a robust SERS sensor for the quantification of thiabendazole fungicide in drinking water. Interestingly, the plasmonic substrate was protected with polymer films as a proof-of-concept for developing more stable and wearable sensors for on-site monitoring. Detection of the pesticide thiram was achieved employing polydopamine spheres coated with a gold shell bearing gaps and voids (hotspots) (Chen et al., 2018). The nanowaxberry substrate (see Figure 1B) achieved a LOD of 2.4 μg/L in spiked environmental water (river water). As the concentration of chemical pollutants in environmental waters is typically in the ng/L to μg/L range (Neale et al., 2018), a pre-concentration step is often required prior to analysis. In this context, plasmonic substrates assembled on filter membranes offer new possibilities for preconcentration and simultaneous detection. Thus, composites of silver NPs and a liquid crystal (LC) polymer supported on polyamide filters has been recently fabricated by Fateixa et al. (2018b) for the extraction and detection of thiram spiked in river samples at 240.4 ng/L. The same group developed a filtering SERS sensor based on polyamide-based composites loaded with plasmonic nanoparticles by filtration (Fateixa et al., 2018a). This SERS-active flexible membrane trapped and concentrated chemical and water pollutants demonstrating detection of crystal violet dye spiked in estuary water samples up to 4.1 pg/L. This work is based on the filter SERS assay originally developed by Yu and White (2012) who reported the field-based application of such sensor for the quantitative detection of ppb concentrations of melamine, a food contaminant, as well as malathion, a widely used organophosphate pesticide, in water. The performance of this SERS assay was up to two orders of magnitude better than the conventional approach of drying a silver colloid onto a surface. In contrast to other solid supports, the three-dimensional structure of paper-based substrates allows both high specific surface and plasmon coupling, further enhancing the SERS signal. The different methods used to fabricate paper and cellulose-based SERS sensors have been recently reviewed elsewhere (Ogundare and van Zyl, 2019; Restaino and White, 2019).

The feasibility for the detection of antibiotics in environmental aquatic samples has been addressed in recent studies (Han et al., 2014; Hidi et al., 2016; Hong et al., 2017; Patze et al., 2017; Fang et al., 2019). Han et al. (2014) used SERS-active Ag nanorod arrays fabricated by oblique angle deposition to detect metronidazole and ronidazole in spiked samples from tap water, lake water, and swamp water. Despite both molecules could be readily detected at μg/mL concentrations regardless of the sample complexity, the LOD in terms of bulk concentrations does not meet mainstream analytical techniques such as liquid chromatography-mass spectrometry (LC-MS), with LODs as low as sub-mg/L. In another study, Ag arrays embedded in a microfluidic system were employed for the SERS detection of the antibiotic sulfamethoxazole down to 0.56 μg/L in spiked aquatic samples (Patze et al., 2017). The combination of a microfluidic setup with a top-down SERS substrate contributed to robust measurement conditions. Interestingly, the multiplex detection and quantification of the antibiotic enrofloxacin and its metabolite ciprofloxacin to mg/L-level in bi-analyte mixtures was demonstrated through the use of Ag nanogratings fabricated using laser interference lithography (Hong et al., 2017).

A big issue in pollutant analysis is the quantification at ultralow concentrations. Although single-molecule SERS (SM-SERS) is a potential tool for ultrasensitive detection, the strong signal fluctuations at ultralow concentration regimes often limit its expectations as a quantitative analytical technique. Recently, de Albuquerque et al. (2018) developed a procedure based on SM-SERS statistics for ultralow concentration quantification without the need for preconcentration. Thus, signals generated by SM-SERS events are “digitized” (i.e., digital SERS; Dos Santos et al., 2019) and the number of pixels within a given mapping area that provides a SM-SERS response (SERS digital count) could be correlated with the solution concentration (see Figure 1C). Finally, the total digital counts recorded for each concentration by SERS mapping were used to generate a calibration curve that enabled to detect enrofloxacin and ciprofloxacin with a low limit of quantification (LOQ) of 1.0 and 0.9 ng/L, respectively. Recently, an aptamer-based conformation cooperated enzyme-assisted SERS technology has been developed for sensitive and high selective detection of antibiotics in trace amounts (Fang et al., 2019). It is based on the enzymatic conversion of the antibiotic to a nucleic acid probe containing a Raman active molecule that is detectable by SERS with high sensitivity. It was demonstrated for chloramphenicol achieving a LOD of 4.8 pg/L in aqueous solution. Another interesting issue is the development of analytical recyclable tools for sensitive detection of environmental contaminants. A proof-of-principle study for recyclable SERS platforms for detection and degradation of the antibiotic tetracycline hydrochloride and the Rhodamine 6G dye was recently developed by Qu et al. (2019). Interestingly, the substrate (graphitic carbon on Ag nanorod arrays) shows self-cleaning abilities under visible light irradiation and could be further reused.

The detection efficiency of pollutants in environmental aquatic samples can be significantly improved employing non-wetting phenomena to concentrate analyte molecules within SERS-active regions (Lee et al., 2019). For instance, a superhydrophobic platform was used to concentrate Rhodamine 6G along with plasmonic nanoparticles within an evaporating liquid droplet, thus enabling to detect this environmentally hazardous dye down to 35.9 fg/L (Yang et al., 2016). With the aim of developing an effective approach for separating oil/water mixture, detecting, and degrading pollutants simultaneously, Xu et al. (2019) reported a superhydrophobic Au/AgCl-coated copper mesh which can separate and solely detect methylene blue (MB) molecules in Sudan III/MB oil/water mixture. After separation, MB can be further photodegraded by the Au/AgCl-coated copper mesh, suggesting its potential application for wastewater treatment.

Owing to their tunable porous structure and excellent adsorption capacity, metal organic-frameworks (MOFs) have been explored as potential adsorbents for aqueous-phase sorptive removal of emerging environmental contaminants (Dhaka et al., 2019). MOFs have been combined with plasmonic nanostructures to create novel detection systems for the selective molecule diffusion at nanoparticle surfaces (Zheng et al., 2016). By tuning the pore size of plasmonic MOFs it is possible to effectively generate sieving effects, thereby reducing potential interferences arising from the biological matrices during SERS measurements. Recently, Au NPs embedded within MIL-101 demonstrated good sensing capabilities for the quantitative analysis of p-phenylenediamine in environmental water achieving a LOD of 0.10 ng/mL (Hu et al., 2014). Similarly, plasmonic MOF nanocomposites consisting of MOF-199, Uio-66, and Uio-67 with encapsulated gold NPs have been applied for the detection of acetamiprid pesticide with 4.4, 2.0, and 4.4 μg/L LODs, respectively (Cao et al., 2017). Interestingly, MOFs can also act as the host to capture targets through their unique porous structures. This ability was used to detect elusive target analytes lacking metal-affinity groups in water (Choi et al., 2019). In this framework, core-shell HKUST-1@AgNP composites demonstrated good sensing capabilities for polycyclic aromatic hydrocarbons (PAHs) in environmental samples, while preserving the cyclability and selectivity required for reliable quantitative analysis (Li et al., 2019). Remarkably, the performance of this plasmonic composite was compared with that of gas chromatography-mass spectrometry (GC-MS) showing similar detection capabilities, suggesting its potential for on-site detection of these pollutants. Focused on PAHs, host-guest approaches based on the use of pillar[5]arenes have been developed for their quantitative, label-free and multiplex SERS detection (Montes-Garcia et al., 2014, 2017). The AP[5]A exhibits excellent properties for pollutant adsorption from water, trapping non-polar molecules through hydrophobic and π-π interactions (Lan et al., 2017). Recyclable AP[5]A-based supramolecular plasmonic thin films enable the reliable quantification of pyrene, nitropyrene, and anthracene in water, as well as the simultaneous detection of the PAHs in a mixture employing chemometrics (see Figure 1D; Montes-Garcia et al., 2017) In a different approach, a substrate made of arrays of gold nanorods functionalized with diazonium salt quantified benzo[a]pyrene, fluoranthene, and naphthalene in water-methanol samples. PAHs were detected via SERS using Au NPs coated with polydopamine (PDA) (Du and Jing, 2019). Interestingly, PDA acted as a reactive scaffold for locking PAHs [phenanthrene, pyrene, benzo[b]fluoranthene, benzo[a]pyrene, and benzo[g,h,i]perylene] into the hotspots for SERS sensing, thereby reaching LOD ranging from 10 to 90 μg/L depending on the PAH (Tijunelyte et al., 2017). This study also showed the identification of the three analytes in the mixture. Recently, a molecularly imprinted polymer (MIP) thin film was combined with Au NP assemblies for SERS recognition of PAHs, such as pyrene or fluoranthene, in the sub μg/L regime (Castro-Grijalba et al., 2020). The role of MIP was to trap the PAH close to the Au surface. The detection of pyrene in creek water and seawater was demonstrated.

In another study, Tu et al. (2019) developed a SERS-based aptasensor for trace analysis of diethylhexylphthalate (DEHP) in tap water, bottled water, and a carbonated beverage employing magnetic particles functionalized with a DEHP aptamer The reported approach showed a detection range from 0.003 to 71 μg/L and a LOD of 3.1 ng/L. In this context, the use of magnetic nanoparticles for SERS detection of environmental pollutants has been recently reviewed (Pinheiro et al., 2018; Song et al., 2019).

## SERS Detection of Inorganic Pollutants

Toxic anions (e.g., nitrite, nitrate, perchlorate ions) and heavy metal (arsenic, mercury, lead, chromium, cadmium, and copper) cations are major environmental contaminants. With the aim to quantify trace amounts of such possible contaminants, environmental monitoring has generated a need for innovative and improved approaches with ever-increasing sensitivity and selectivity for the detection of these hazardous chemical species. In general, oxyanions, especially those with moderate Raman cross-sections (e.g., perchlorate) can be detected by their vibrational signatures. In contrast, direct SERS detection of monatomic metal ions is more challenged due to their small scattering cross-section (Tang et al., 2018).

Sensitive detection of perchlorate anions by SERS relies on the surface functionalization of the plasmonic material with positively charged reagents such as cystamine, 2-dimethyl-aminoethanethiol, or poly(ethyleneimine) (Hao and Meng, 2017; Jubb et al., 2017). Stewart et al. (2015) reported a colloidal detection approach for nitrate and perchlorate, as target analytes, based on formation of hotspots through NaCl induced aggregation of quaternary ammonium-terminated thiocholine stabilized silver colloids. In a recent study, SERS substrates based on gold ellipse dimers functionalized with 2-(dimethylamino)ethane-thiol were used to detect and quantify ${\text{ClO}}_{4}^{-}$ contamination at the μg/L level within groundwaters, thereby demonstrating the applicability of this approach for field measurements (Jubb et al., 2017).

**Figure 2:**
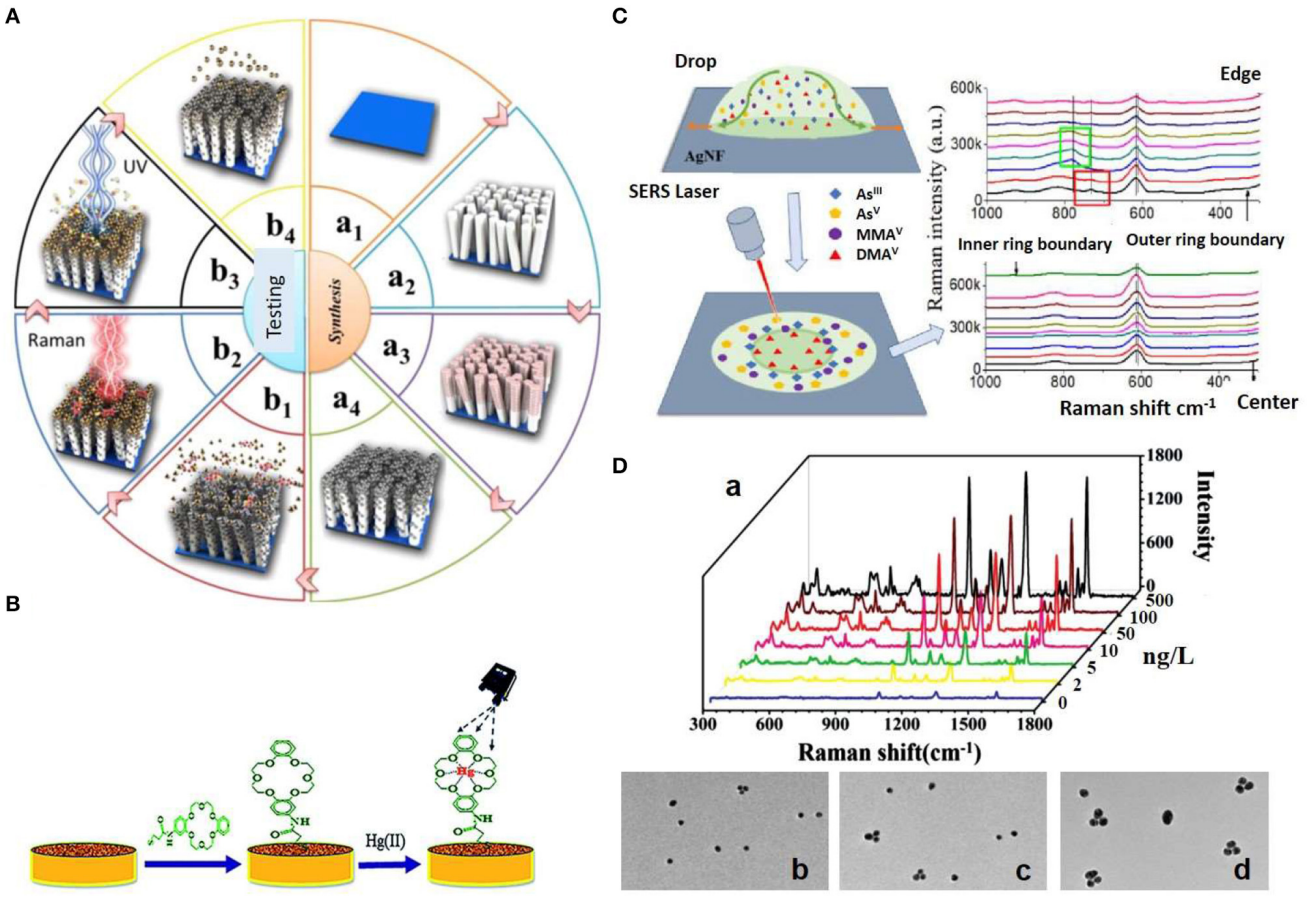
Strategies for SERS detection of inorganic pollutants. **(A)** Schematic illustration of the fabrication of ZnO/Ag nanoarrays for the Hg^2+^ ions SERS detection. (a) The ZnO/Ag nanoarrays were fabricated by growing ZnO nanoarrays via soft hydrothermal method (a1, a2) and the subsequent deposition of Ag nanoparticles via an electroless plating technique (a3, a4). (b) Adsorption of Hg^2+^ ions and subsequent adsorption of Rhodamine B (RB) on the on ZnO/Ag nanoarrays (b1), Hg^2+^ ions detection via SERS monitoring of RB (b2), photocatalytic degradation of RB (b3), and Hg^2+^ removal via heat treatment (b4). Reproduced from Esmaielzadeh Kandjani et al. (2015) with permission from American Chemical Society (Copyright 2015). **(B)** Schematic representation of the modification of nanostructured Au substrate with a crown ether derivative for capturing Hg^2+^. Reproduced from Sarfo et al. (2017) with permission from The Royal Society of Chemistry. **(C)** SERS method for arsenic speciation by using the separation potential of the coffee ring effect on negatively charged Ag nanofilms. Reproduced from Yang et al. (2019) with permission from American Chemical Society (Copyright 2019). **(D)** Triple Raman label-encoded Au NPs trimer for heavy metal ion detection. (a) SERS spectra under the same concentration of Hg^2+^ and Ag^+^ ranging from 0 to 500 ng/L. (b–d) TEM images of Au NP trimers assembled by the addition of equal concentration of Hg^2+^ and Ag^+^ at different concentrations: 5 (b), 50 (c), and 100 (d) ng/L. Reproduced from Li et al. (2015) with permission from John Wiley & Sons (Copyright 2015).

Arsenic species, including arsenate (As^5+^) and arsenite (As^3+^), which usually exist in the environment as ${\text{AsO}}_{4}^{3-}$ or ${\text{AsO}}_{3}^{3-}$, respectively, can be directly detected by SERS based on the characteristic vibration of As–O stretch mode (Hao et al., 2015). However, since the Raman cross-sections of these inorganic oxyanions are not large and their affinity to metallic surfaces is remarkably low, most reported strategies for the detection of heavy metal cations or oxyanions usually follow indirect approaches. For instance, the surface of SERS substrates can be functionalized with a positively charged layer to enhance the affinity of the metal cation toward the plasmonic surface. In a different approach, the detection takes advantage of the affinity between the metal cation and a Raman active component of the plasmonic substrate. In this context, Wang et al. (2013) fabricated core-shell Ag@polyaniline nanocomposites as active SERS nanoprobes for the detection of Hg^2+^ ions with a detection limit of 0.2 ng/L. The analysis of the intensity changes of the SERS signal from polyaniline at 1,560 cm^−1^ was shown to be strongly dependent on the concentrations of Hg^2+^. Carbon nanotubes over a porous anodic alumina membrane were used for the trace detection of Hg^2+^, Cd^2+^, and Pb^2+^ (Shaban and Galaly, 2016). Du et al. (2013) fabricated a sensing system consisting of core-shell Au@Ag NPs and an organic ligand 4,4′-Dipyridyl (Dpy) for Hg^2+^ sensing. This molecule induced the aggregation of the NPs, generating strong Raman hotspots and SERS readouts. As Hg^2+^ shows a high affinity toward Dpy, it can inhibit the aggregation of Au@Ag NPs, thus quenching the SERS signal from Dpy. This colloidal-based approach demonstrated high sensitivity, detecting Hg^2+^ residues at the pg/L level, and specificity toward mercury, as it was not responsive to other metal ions tested. Kandjani reported a SERS-active thin film of ZnO/Ag nanoarrays for Hg^2+^ detection. In this study, the change in intensity of the characteristic Raman peak of Rhodamine B at 1,358 cm^−1^ was used for detection and quantification of the Hg^2+^ ions in solution. Additionally, the photocatalytic activity of the nanoarrays allowed the removal of mercury, and reusability of the substrate over many cycles (see Figure 2A; Esmaielzadeh Kandjani et al., 2015). This sensing device showed a limit of detection in the sub ppb range, and high selectivity toward Hg^2+^. In another study, a crown ether derivative (TCE) was self-assembled onto the surface of a nanostructured gold substrate for Hg^2+^ sensing (Sarfo et al., 2017). The coordination of Hg^2+^ to the oxygen atoms of TCE could be monitored by SERS, thereby enabling the detection of mercury in tap water at toxic concentration of 3.35 μg/L using a handheld Raman spectrometer (see Figure 2B).

Accurate analysis of toxic metals and metalloids such as arsenic requires to maintain the integrity of the often labile chemical species, which is often impaired during sample preparation, separation, or detection. In this framework, Yang et al. reported a novel SERS method for arsenic speciation by combining the separation potential of the coffee ring effect on negatively charged silver nanofilms (AgNFs) for the detection of four common arsenic species, ${\text{AsO}}_{4}^{3-}$, ${\text{AsO}}_{3}^{3-}$, monomethylarsonic acid (MMA^V^), and dimethylarsinic acid (DMA^V^) (see Figure 2C; Yang et al., 2019). The combined interactions of arsenic species with the AgNFs, solvent, and sodium dodecyl sulfate surfactant, made possible arsenic speciation and SERS detection at 0.1 μg/L, demonstrating the potential of this approach for rapid separation and qualitatively SERS analysis. Toward quantitative analysis of Hg^2+^ speciation into methylmercury (CH_3_Hg^+^), Guerrini et al. (2014) fabricated a sensing platform consisting 4-mercaptopyridine (MPY) functionalized Au NPs anchored onto polystyrene microbeads. The co-ordination of Hg^2+^ and CH_3_Hg^+^ to the nitrogen atom of the MPY ring yields characteristic changes in the vibrational SERS spectrum of MPY that can be qualitatively and quantitatively correlated with the presence of the two different mercury species. Thus, in aqueous samples a limit of detection of 1.5 and 0.1 μg/L was achieved for CH_3_Hg^+^ and Hg^2+^, respectively, when a concentration of beads in solution of 0.8 mg/mL was used.

Simultaneous detection and quantification of different inorganic pollutants is an important asset. Li et al., demonstrated a stable and reliable SERS method for multiplex detection of Hg^2+^ and Ag^+^ using triple Raman-encoded Au NP trimers with LODs of 3.4 and 0.92 ng/L, respectively (see Figure 2D; Li et al., 2015) Thus, the presence of Hg^2+^ and/or Ag^+^ induced the assembly of the Au NPs into trimers producing enhancements of the Raman reporters encoding the NPs. Recently, Cd^2+^ ions were detected by using Au NPs functionalized with dopamine quinone (DQ) (Du and Jing, 2019). The strong binding affinity of DQ toward Cd^2+^ facilitates the entrapment of the ions close to the Au surface, allowing its qualitative determination with a detection limit of 1.1 μg/L. Finally, SERS has also applied for the detection of fluorosurfactants in aqueous solution and in spiked groundwater (Fang et al., 2016).

## Challenges and Outlook

In this minireview we have summarized recent approaches for SERS detection of organic and inorganic chemical pollutants in aqueous media. As shown herein, a wide variety of strategies for detecting analytes have been adopted to potentially overcome the limitations in SERS sensing. SERS can enhance the sensitivity and selectivity of chemical detection, reducing the analytical time, sample consumption, as well as facilitating miniaturization and on-site analysis with portable Raman devices. All these rapid advances offer a bright future for SERS. Nevertheless, although great progress has been made, many challenges still remain for a realistic implementation of SERS in environmental analysis where analytes of diverse nature are to be detected: (i) optical and chemical properties of SERS platforms should be further optimized; (ii) the reproducibility in the synthesis of the SERS substrates in different batches and from different labs is still an issue; (iii) the lack of standardized protocols does not facilitate the comparison of electromagnetic enhancement factors between different laboratories; (iv) most of these new sensing tools are validated only in the lab with model analytes characterized by high Raman cross-section and not actual pollutants; (v) despite the delicate design and finely tuning of SERS substrates their performance in real samples is usually not as good as expected, mainly due to an insufficient consideration of environmental factors that can influence the measurements; (vi) it is also desirable to develop sensing approaches with reusability and recyclability capabilities to make them more cost-effective. Finally, many factors influence the SERS signal such as the strength of the local electromagnetic field, the nature of the analyte, its concentration, the chemical affinity to the nanoparticle surface, as well as the stability of the analyte-nanoparticle among others. Whereas, the interplay between these factors opens up a wide range of possibilities, the rational design of the plasmonic substrate for a specific application is often mandatory, thereby limiting the generalization of its use.

The broad interest in SERS together with the improved control over substrate fabrication, as well as the development of new related-instrumentation has resulted in the continuous development of advanced plasmonic platforms (e.g., chemosensors, chiral-selective systems, SHINERS, intragap core–shell particles), as well as emerging surface-enhanced signal amplification techniques (e.g., TERS, SEIRA, EC-SERS, SESORRS, etc.; Langer et al., 2019). These advancements offer immensely attractive approaches to potentially overcome the limitations in SERS sensing, which will eventually aid to bring this powerful technology out of the laboratory into real world applications. Thus, despite current challenges we envision that this spectroscopic technique will soon become a widespread analytical tool for routine monitoring of environmental waters and wastewater treatment plants in the near future.

## Author Contributions

GB and IP-S have contributed equally to the writing process of this minireview.

## Conflict of Interest

The authors declare that the research was conducted in the absence of any commercial or financial relationships that could be construed as a potential conflict of interest.
