# Supplementary material for: Recent Progress in Surface-Enhanced Raman Scattering for the Detection of Chemical Contaminants in Water
Source: Front Chem. 2020 Jun 9;8:478. doi: 10.3389/fchem.2020.00478 (PMC7296159; doi:10.3389/fchem.2020.00478)
Supplement: Supplementary file 1 [file Table_1.pdf]

Supporting Information

Recent progress in surface-enhanced Raman scattering for the detection of chemical contaminants in water

Gustavo Bodelón[1,2]* and Isabel Pastoriza-Santos[1,2]*

[1] CINBIO, Universidade de Vigo, Vigo, Spain
[2] Galicia Sur Health Research Institute (IIS Galicia Sur), SERGAS-UVIGO, Vigo, Spain

*** Correspondence:**
Dr. Gustavo Bodelón
gbodelon@uvigo.es

Dr. Isabel Pastoriza-Santos
pastoriza@uvigo.es

Keywords: plasmonic nanostructures, SERS, sensing, chemical contaminants, water

Table 1. Summary of current approaches undertaken of different SERS sensing approaches for organic and inorganic pollutants in water.

Contaminant	SERS substrate	Sensitivity	Sample	Reference
DDT	SiO_2 capsules innerly doped with AuNPs	1.77 µg/L	RW	(Marino-Lopez, Sousa-Castillo et al. 2019)
Thiabendazole	Film-packaged Au/Ag bimetallic chip	20 µg/L	DW^S	(Wang, Sun et al. 2019)
Thiram	Core-shell polydopamine-Au beads	2.4 µg/L	RW^S	(Chen, Zhu et al. 2018)
Thiram	AgNPs-LC composites on polyamide filter	240.4 ng/L	RW^S	(Fateixa, Raposo et al. 2018)
Crystal violet	AgNPs doped polyamide membranes	4.1 pg/L	ESW^S	(Fateixa, Nogueira et al. 2018)
Metronidazole<br>Ronidazole	Ag nanorod arrays	10 mg/L<br>50 mg/L	EW^S	(Han, Chen et al. 2014)
Sulfamethoxazole	Ag arrays in microfluidics	0.56 µg/L	TW^S	(Patze, Huebner et al. 2017)
Enrofloxacin<br>Ciprofloxacin	Ag nanogratings	359.4 mg/L<br>331.3 mg/L	AS	(Hong, de Albuquerque et al. 2017)
Chloramphenicol	AuNPs@Si substrates	4.8 pg/L	AS	(Fang, Li et al. 2019)
Enrofloxacin<br>Ciprofloxacin	AuNPs over coverslip glass	1.0 ng/L<br>0.9 ng/L	AS	(de Albuquerque, Sobral-Filho et al. 2018)
Tetracycline	3D AgNRs/O-g-C_3N_4 substrates	44.4 µg/L	AS	(Qu, Geng et al. 2019)
Rhodamine 6G	AuNPs /SLIPSERS platform	35.9 fg/L	AS	(Yang, Dai et al. 2016)
Phenylenediamine	AuNPs/MIL-101 MOF	100 ng/L	RW, SW, SEW^s	(Hu, Liao et al. 2014)
Acetamiprid	AuNPs/MOFs	4.4-2.0 µg/L	AS	(Cao, Hong et al. 2017)
Antracene/Pyrene/ Perylene/4-Chlorobiphenyl	AgNPs/ HKUST-1 MOF	3,5/0.03/0.7 /0.94 µg/L	AS	(Li, Cao et al. 2019)
Antracene/ Nitropyrene/Pyrene	pillar[5]arene-AuNPs thin films	0.17/0.25 µg/L /2 ng/L	AS	(Montes-Garcia, Gomez-Gonzalez et al. 2017)
Pyrene/fluoroanthene	MIPs-AuNPs thin films		CW, SW	(Castro-Grijalba 2020)
Diethylhexylphthalate	AgNPs aptasensor	3.1 ng/L	TW^S	(Tu, Garza et al. 2019)
ClO_4^-	Au ellipse dimer arrays	26 µg/L	GW	(Jubb, Hatzinger et al. 2017)
Hg^{2+}	Ag@polyaniline NPs	0.2 ng/L	AS	(Wang, Shen et al. 2013)
Hg^{2+}, Cd^{2+}, Pb^{2+}	CNTs/$CoFe_2O_4$ composite	1.0 µg/L	AS	(Shaban and Galaly 2016)

Hg^{2+}	4,4′-Dipyridyl Au@Ag NPs	2.0 pg/L	AS	(Du, Liu et al. 2013)
Hg^{2+}	ZnO/Ag nanoarrays	0.45 µg/L	AS	(Esmaielzadeh Kandjani, Sabri et al. 2015)
Hg^{2+}	Crown-ethers modified Au nanostructures	3.35 µg/L	TW^{S}	(Sarfo, Sivanesan et al. 2017)
Arsenic species	Ag nanofilms	0.1 µg/L	AS	(Yang, Liamtsau et al. 2019)
Hg^{2+}/CH_3Hg^{+}	AuNPs-doped polystyrene beads	0.1/1.5 µg/L	AS	(Guerrini, Rodriguez-Loureiro et al. 2014)
Hg^{2+}/Ag^{+}	Raman-encoded AuNP trimers	3.4/0.92 ng/L	AS	(Li, Xu et al. 2015)
Cd^{2+}/PAHs	Polydopamine-coated Au NPs	1.1 /10-90 µg/L	AS	(Du and Jing 2019)
Fluorosurfactant	Ag colloids on graphene oxide	50 µg/L	GW^{S}	(Fang, Megharaj et al. 2016)

(^{S})-Spyked; RW-River Water; DW-Drinking Water; SW-sea water; CW-creek water; AS-Aqueous Solution; GW-Ground Water; ESW- Estuary Seawater; SEW-sewage water; EW-environmental water

Cao, X., S. Hong, Z. Jiang, Y. She, S. Wang, C. Zhang, H. Li, F. Jin, M. Jin and J. Wang (2017). "SERS-active metal-organic frameworks with embedded gold nanoparticles." Analyst **142**(14): 2640-2647.

Castro-Grijalba, A. M.-G., V; Cordero-Ferradás, M.J.; Coronado, E. A.; Perez-Juste, J; and Pastoriza-Santos, I. (2020). "SERS-Based Molecularly Imprinted Plasmonic Sensor for Highly Sensitive PAH Detection." ACS Sensors(5): 693-702.

Chen, D. Z., X. D. Zhu, J. Huang, G. Wang, Y. Zhao, F. Chen, J. Wei, Z. X. Song and Y. X. Zhao (2018). "Polydopamine@Gold Nanowaxberry Enabling Improved SERS Sensing of Pesticides, Pollutants, and Explosives in Complex Samples." Analytical Chemistry **90**(15): 9048-9054.

de Albuquerque, C. D. L., R. G. Sobral-Filho, R. J. Poppi and A. G. Brolo (2018). "Digital Protocol for Chemical Analysis at Ultralow Concentrations by Surface-Enhanced Raman Scattering." Anal Chem **90**(2): 1248-1254.

Du, J. and C. Jing (2019). "One-step fabrication of dopamine-inspired Au for SERS sensing of Cd(2+) and polycyclic aromatic hydrocarbons." Anal Chim Acta **1062**: 131-139.

Du, Y., R. Liu, B. Liu, S. Wang, M. Y. Han and Z. Zhang (2013). "Surface-enhanced Raman scattering chip for femtomolar detection of mercuric ion (II) by ligand exchange." Anal Chem **85**(6): 3160-3165.

Esmaielzadeh Kandjani, A., Y. M. Sabri, M. Mohammad-Taheri, V. Bansal and S. K. Bhargava (2015). "Detect, remove and reuse: a new paradigm in sensing and removal of Hg (II) from wastewater via SERS-active ZnO/Ag nanoarrays." Environ Sci Technol **49**(3): 1578-1584.

Fang, C., M. Megharaj and R. Naidu (2016). "Surface-enhanced Raman scattering (SERS) detection of fluorosurfactants in firefighting foams." Rsc Advances **6**(14): 11140-11145.

Fang, Q., Y. Li, X. Miao, Y. Zhang, J. Yan, T. Yu and J. Liu (2019). "Sensitive detection of antibiotics using aptamer conformation cooperated enzyme-assisted SERS technology." Analyst **144**(11): 3649-3658.

Fateixa, S., H. I. S. Nogueira and T. Trindade (2018). "Surface-Enhanced Raman Scattering Spectral Imaging for the Attomolar Range Detection of Crystal Violet in Contaminated Water." Acs Omega **3**(4): 4331-4341.

Fateixa, S., M. Raposo, H. I. S. Nogueira and T. Trindade (2018). "A general strategy to prepare SERS active filter membranes for extraction and detection of pesticides in water." Talanta **182**: 558-566.

Guerrini, L., I. Rodriguez-Loureiro, M. A. Correa-Duarte, Y. H. Lee, X. Y. Ling, F. J. Garcia de Abajo and R. A. Alvarez-Puebla (2014). "Chemical speciation of heavy metals by surface-enhanced Raman scattering spectroscopy: identification and quantification of inorganic- and methyl-mercury in water." Nanoscale **6**(14): 8368-8375.

Han, C. Q., J. Chen, X. M. Wu, Y. W. Huang and Y. P. Zhao (2014). "Detection of metronidazole and ronidazole from environmental Samples by surface enhanced Raman spectroscopy." Talanta **128**: 293-298.

Hong, K. Y., C. D. L. de Albuquerque, R. J. Poppi and A. G. Brolo (2017). "Determination of aqueous antibiotic solutions using SERS nanogratings." Analytica Chimica Acta **982**: 148-155.

Hu, Y., J. Liao, D. Wang and G. Li (2014). "Fabrication of gold nanoparticle-embedded metal-organic framework for highly sensitive surface-enhanced Raman scattering detection." Anal Chem **86**(8): 3955-3963.

Jubb, A. M., P. B. Hatzinger and B. Gu (2017). "Trace-level perchlorate analysis of impacted groundwater by elevated gold ellipse dimer nanoantenna surface-enhanced Raman scattering." Journal of Raman Spectroscopy **48**(4): 518-524.

Li, D., X. K. Cao, Q. M. Zhang, X. G. Ren, L. Jiang, D. W. Li, W. Deng and H. T. Liu (2019). "Facile in situ synthesis of core-shell MOF@Ag nanoparticle composites on screen-printed electrodes for ultrasensitive SERS detection of polycyclic aromatic hydrocarbons." Journal of Materials Chemistry A **7**(23): 14108-14117.

Li, S., L. Xu, W. Ma, H. Kuang, L. Wang and C. Xu (2015). "Triple Raman Label-Encoded Gold Nanoparticle Trimers for Simultaneous Heavy Metal Ion Detection." Small **11**(28): 3435-3439.

Marino-Lopez, A., A. Sousa-Castillo, M. Blanco-Formoso, L. N. Furini, L. Rodriguez-Lorenzo, N. Pazos-Perez, L. Guerrini, M. Perez-Lorenzo, M. A. Correa-Duarte and R. A. Alvarez-Pueble (2019). "Microporous Plasmonic Capsules as Stable Molecular Sieves for Direct SERS Quantification of Small Pollutants in Natural Waters." Chemnanomat **5**(1): 46-50.

Montes-Garcia, V., B. Gomez-Gonzalez, D. Martinez-Solis, J. M. Taboada, N. Jimenez-Otero, J. de Una-Alvarez, F. Obelleiro, L. Garcia-Rio, J. Perez-Juste and I. Pastoriza-Santos (2017). "Pillar[5]arene-Based Supramolecular Plasmonic Thin Films for Label-

Free, Quantitative and Multiplex SERS Detection." ACS Appl Mater Interfaces **9**(31): 26372-26382.

Patze, S., U. Huebner, F. Liebold, K. Weber, D. Cialla-May and J. Popp (2017). "SERS as an analytical tool in environmental science: The detection of sulfamethoxazole in the nanomolar range by applying a microfluidic cartridge setup." Analytica Chimica Acta **949**: 1-7.

Qu, L. L., Z. Q. Geng, W. Wang, K. C. Yang, W. P. Wang, C. Q. Han, G. H. Yang, R. Vajtai, D. W. Li and P. M. Ajayan (2019). "Recyclable three-dimensional Ag nanorod arrays decorated with O-g-C3N4 for highly sensitive SERS sensing of organic pollutants." J Hazard Mater **379**: 120823.

Sarfo, D. K., A. Sivanesan, E. L. Izake and G. A. Ayoko (2017). "Rapid detection of mercury contamination in water by surface enhanced Raman spectroscopy." Rsc Advances **7**(35): 21567-21575.

Shaban, M. and A. R. Galaly (2016). "Highly Sensitive and Selective In-Situ SERS Detection of Pb(2+), Hg(2+), and Cd(2+) Using Nanoporous Membrane Functionalized with CNTs." Sci Rep **6**: 25307.

Tu, D., J. T. Garza and G. L. Cote (2019). "A SERS aptasensor for sensitive and selective detection of bis(2-ethylhexyl) phthalate." RSC Adv **9**(5): 2618-2625.

Wang, K., D. W. Sun, H. Pu, Q. Wei and L. Huang (2019). "Stable, Flexible, and High-Performance SERS Chip Enabled by a Ternary Film-Packaged Plasmonic Nanoparticle Array." ACS Appl Mater Interfaces **11**(32): 29177-29186.

Wang, X. F., Y. H. Shen, A. J. Xie and S. H. Chen (2013). "One-step synthesis of Ag@PANI nanocomposites and their application to detection of mercury." Materials Chemistry and Physics **140**(2-3): 487-492.

Yang, M., V. Liamtsau, C. Fan, K. L. Sylvers, A. J. McGoron, G. Liu, F. Fu and Y. Cai (2019). "Arsenic Speciation on Silver Nanofilms by Surface-Enhanced Raman Spectroscopy." Anal Chem **91**(13): 8280-8288.

Yang, S., X. Dai, B. B. Stogin and T. S. Wong (2016). "Ultrasensitive surface-enhanced Raman scattering detection in common fluids." Proc Natl Acad Sci U S A **113**(2): 268-273.
